# Using bioacoustics to examine shifts in songbird phenology

**DOI:** 10.1002/ece3.2242

**Published:** 2016-06-12

**Authors:** Rachel T. Buxton, Emma Brown, Lewis Sharman, Christine M. Gabriele, Megan F. McKenna

**Affiliations:** ^1^ Department of Fish, Wildlife and Conservation Biology Colorado State University 1474 Campus Delivery Fort Collins Colorado 80523; ^2^ Natural Sounds and Night Skies Division National Park Service 1201 Oakridge Drive Fort Collins Colorado 80525; ^3^ Glacier Bay National Park and Preserve PO Box 140 Gustavus Alaska 99826

**Keywords:** Acoustic Complexity Index, Alaska, climate change, Glacier Bay, monitoring, songbirds, soundscape

## Abstract

Monitoring patterns in biodiversity and phenology have become increasingly important given accelerating levels of anthropogenic change. Long‐term monitoring programs have reported earlier occurrence of spring activity, reflecting species response to climate change. Although tracking shifts in spring migration represents a valuable approach to monitoring community‐level consequences of climate change, robust long‐term observations are challenging and costly. Audio recordings and metrics of bioacoustic activity could provide an effective method for monitoring changes in songbird activity and broader biotic interactions. We used 3 years of spring and fall recordings at six sites in Glacier Bay National Park, Alaska, an area experiencing rapid warming and glacial retreat, to examine the utility of bioacoustics to detect changes in songbird phenology. We calculated the Acoustic Complexity Index (ACI), an algorithm representing an index of bird community complexity. Abrupt changes in ACI values from winter to spring corresponded to spring transition, suggesting that ACI may be an effective, albeit coarse metric to detect the arrival of migrating songbirds. The first peak in ACI shifted from April 16 to April 11 from 2012 to 2014. Changes in ACI were less abrupt in the fall due to weather events, suggesting spring recordings are better suited to indicate phenology. To ensure changes in ACI values were detecting real changes in songbird activity, we explored the relationship between ACI and song of three species: varied thrush** **(*Ixoreus naevius*), Pacific wren (*Troglodytes pacificus*), and ruby‐crowned kinglet (*Regulus calendula*). ACI was positively related to counts of all species, but most markedly with song of the varied thrush, the most common species in our recordings and a known indicator of forest ecosystem health. We conclude that acoustic recordings paired with bioacoustic indices may be a useful method of monitoring shifts in songbird communities due to climate change and other sources of anthropogenic disturbance.

## Introduction

Identifying and predicting spatiotemporal changes in biodiversity has been identified as a major scientific challenge given the current rate of global change (Magurran and Dornelas 2010; Dornelas et al. 2013). Assessment of patterns in biodiversity requires analytic techniques that can capture the complexity of community and ecosystem dynamics (Magurran 2003, 2011). This can include the use of indicator species (Pearson 1994; Carignan and Villard 2002), rapid biodiversity assessments (Oliver and Beattie 1993; Kerr et al. 2000), or more recently, the use of acoustic surveying (Sueur et al. 2008b; Lellouch et al. 2014). Monitoring biodiversity at this relatively coarse scale may reveal shifts in community dynamics and underlying ecosystem function, thus informing more comprehensive and adaptive conservation management decision‐making (Lambeck 1997).

Phenology is the study of the timing of periodically recurring biological phenomena (Lieth 1974). Phenological changes in migratory patterns of birds have been proposed as a useful indicator of shifts in community structure in response to changing environmental conditions, namely global climate change (Peñuelas and Filella 2001; Parmesan and Yohe 2003). An increasing number of studies from a wide range of regions demonstrate that spring arrival of migrants has occurred progressively earlier since the 1960s (Bradley et al. 1999; Walther et al. 2002; Lemoine and Böhning‐Gaese 2003; Wood and Kellermann 2015). Importantly, the timing of change in different taxonomic groups is not always synchronous and may have profound ecosystem‐level consequences (Walther et al. 2002; Walther 2010).

Earlier arrival and breeding of songbirds have been attributed in large part to increases in spring temperatures (Brown et al. 1999; Lehikoinen et al. 2004; Menzel et al. 2006). Each spring, billions of birds representing hundreds of species migrate north between wintering areas in the United States, Mexico, Central America, and South America to Arctic and subarctic breeding grounds (Newton 2007). Each individual journey can be in excess of 4000 km, with birds using numerous stopover locations along the way, resulting in considerable time and energy cost (Wikelski et al. 2003; Stutchbury et al. 2009). The timing of bird migration is thus determined by a combination of species‐specific endogenous and environmental factors and a myriad of anthropogenic factors (Lack 1960). Habitat fragmentation, depredation by introduced predators, collisions, light pollution, and other anthropogenic threats have resulted in long‐term declines of migratory songbird species and changes in migratory phenology (Loss et al. 2013; Gaston et al. 2014). Because patterns of migratory bird phenology can represent direct indicators of both biospheric changes due to warming and landscape‐level changes occurring along migration routes, patterns of migratory phenology are of particular interest (Root et al. 2003).

Although migratory bird arrival and departure can be conspicuous, its large scale and considerable complexity presents challenges to observation and measurement. Many phenological studies rely on records from local field stations or from historical datasets (Whitfield 2001; Hüppop and Hüppop 2003). Monitoring stations along migration routes use numerous techniques such as mist‐netting, point counts, radar, and more recently, the analysis of night flight calls (Dunn 2005; Farnsworth 2005, 2008). Monitoring migration at a limited number of sites along migration routes limits sampling to birds in the immediate vicinity of census points; thus, estimates may be strongly influenced by subtle changes in migratory routes (Larkin and Szafoni 2008; Sanders and Mennill 2014). Although radar allows for broader spatial scales, species identity within flocks cannot be discerned (Gauthreaux and Belser 2003). Moreover, identification of birds on the basis of flight calls continues to be a major challenge because interspecific calls can show high similarity and intraspecific calls exhibit extensive variability, making species difficult to distinguish (Evans and Rosenberg 2000). Finally, mist‐netting, point counts, and night flight call identification can be biased and labor intensive, often depending on groups of dedicated field personnel who require significant training (Bas et al. 2008; Digby et al. 2013).

Here, we used acoustic recording to examine phenological patterns in the spring and fall soundscape at Glacier Bay National Park and Preserve (GBNP) in southeast Alaska, a stopover and summer breeding ground for numerous migratory bird species. Although acoustic signals have been used for many years to census vocal organisms, acoustic and soundscape ecology are relatively new fields of research (Krause 2002; Celis‐Murillo et al. 2009). The soundscape, defined from an ecological perspective as the geological, biological, and anthropogenic sounds that make up a landscape or seascape, is thought to represent a “footprint of an ecosystem,” reflecting the dynamics of community structure and function (Schafer 1977; National Park Service 2006; Pijanowski et al. 2011a,b). Furthermore, soundscapes can reveal important information about underlying spatial and temporal distributions of vocal species in ecosystems, including changes in diversity, abundance, and behavior (Haselmayer and Quinn 2000; Celis‐Murillo et al. 2009; Farina et al. 2011a). The study of soundscapes has expanded rapidly, driven largely by technological advances in the quality and cost‐effectiveness of recording devices (Blumstein et al. 2011). Moreover, the availability of new metrics, known as acoustic indices, allows the rapid extraction of meaningful biological information from enormous volumes of acoustic data (Sueur et al. 2008b; Kasten et al. 2012; Depraetere et al. 2013; Gasc et al. 2013; Towsey et al. 2014b). Acoustic indices may thus be a fast and effective method of monitoring songbird phenology and hold potential for monitoring broader shifts in ecosystem functioning.

We used acoustic indices to process continuous recordings to monitor the timing of spring and fall songbird migration and characterize ecological complexity. We employed a recently developed acoustic index, the Acoustic Complexity Index (ACI), which is particularly useful for highlighting changes in the behavior and composition of avian communities (Farina et al. 2011b). Our objectives were to: (1) use ACI to indicate changes in winter‐to‐spring and summer‐to‐fall bioacoustic activity; (2) determine whether changes in the bioacoustic activity were reliably predicted by the relative abundance of common migratory songbirds; and (3) examine changes in the acoustic environment between years.

## Materials and Methods

### Acoustic monitoring sites

**Figure 1:**
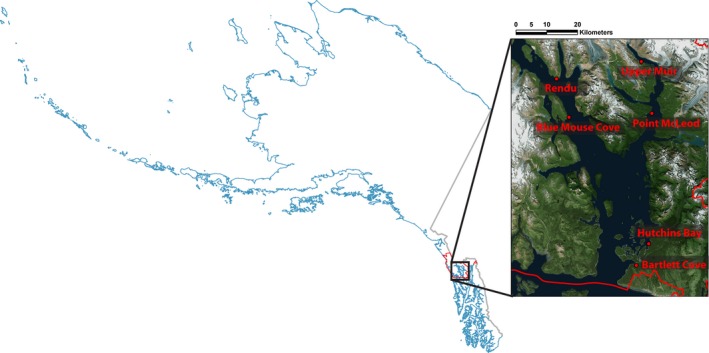
Location of six acoustic recorders in Glacier Bay National Park and Preserve (red outline), southeastern Alaska.

We recorded at six sites within GBNP (58.46°N, 135.86°W, Table 1). Glacier Bay is a glacially carved estuary with one of the highest deglaciation and sedimentation rates in the world, resulting in a dynamic and relatively young ecosystem (Etherington et al. 2007). Glacier Bay supports high marine biological diversity and productivity of birds, marine mammals, fishes, and invertebrates (Robards et al. 2003). Moreover, the bay is surrounded by steep mountainous terrain, resulting in a diversity of terrestrial habitat types (Fig. 1). GBNP, designated as a National Monument in 1925 and a National Park in 1980, includes over 3 million acres and is visited by over 400,000 visitors annually (National Park Service 2008, 2014). Given the high concentration of coastal biodiversity, accelerating habitat alteration due in part to climate change (Clark et al. 2010), and management concern about the impacts of visitation on natural resources, park managers are in the early stages of developing a program to assess soundscape resources.

**Table 1:** Details of six acoustic recording sites in Glacier Bay National Park and Preserve, Alaska

Site name	Recording dates	Vegetation	Elevation	Coordinates
Hutchins Bay	8/24/2011–9/14/2011	Alder/spruce forest	<3 m above high tide	58.5346, −135.8531
Point McLeod	8/19/2011–9/11/2011	Alder/willow thicket	<7 m above high tide	58.9152, −136.11082
Blue Mouse Cove	8/24/2011–9/10/2011	Bare/alder/willow	~10 m	58.79566, −136.490728
Upper Muir	8/19/2011–9/12/2011	Alder/willow thicket	~4 m above high tide	59.0512, −136.26954
Rendu	8/3/2011–8/26/2011	Bare/alder/willow	~5 m	58.88996, −136.63035
Bartlett Cove	2/24/2012–6/2/2012			58.45625, −135.86603
	2/26/2013–6/14/2013			
	3/30/2014–6/10/2014			

### Acoustic recording and analysis

Continuous acoustic recordings were collected using a Roland R‐05 digital audio recorder (Roland, Osaka, Japan) mounted on a tripod at each site. Recordings were collected in the late winter and spring at the mouth of the Bartlett Cove from 2012–2014 and during the late summer and fall of 2011 at five other sites around the bay (Table 1). Each recorder was placed under a weather‐proofing shield to protect microphones from wind and rain (Lynch 2012).

**Figure 2:**
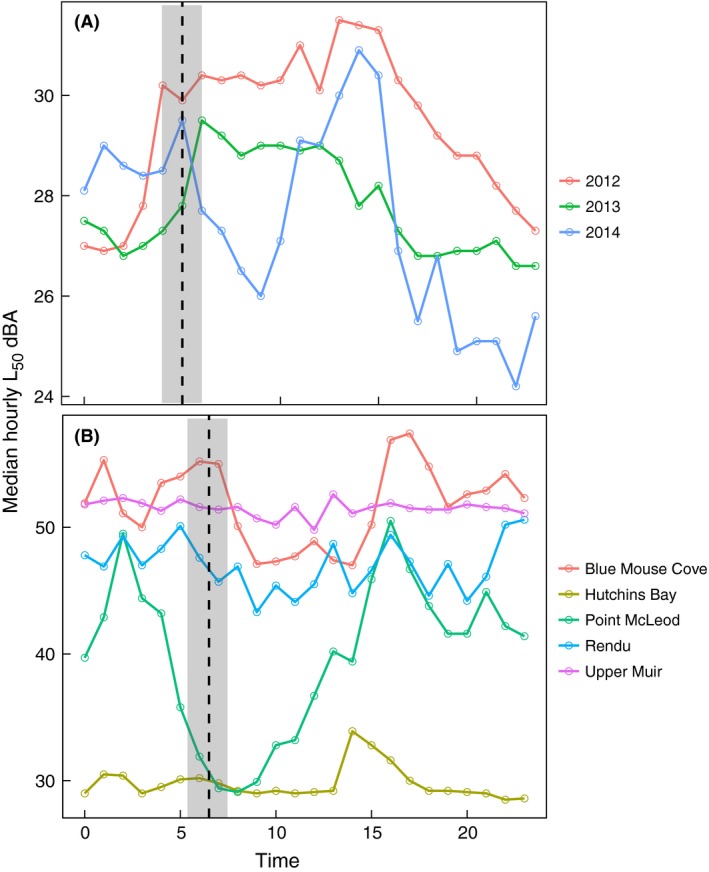
Hourly median dBA values of broadband (12.5–20 kHz) sound pressure levels (median sound pressure level = L_50_) for (A) all data collected in spring (March/April) 2012–2014 at Bartlett Cove and (B) all data collected in fall (September) 2011 at 5 other recording sites in Glacier Bay. Dashed line represents mean monthly sunrise, and shaded areas represent standard deviation.

All audio data were collected in mp3 format at a sampling rate of 44.1 kHz. The audio data were then converted to calibrated 1‐sec 1/3 octave band sound pressure level (SPL) measurements from 12.5–6300 Hz (Mennitt and Fristrup 2012). To explore variation in sound levels among sites and years, we used hourly median levels of broadband A‐weighted SPLs (L_50_) calculated over the entire frequency range across all recording days. Broadband median sound levels represent an average background level including all sources. Because we were interested in sound associated with songbirds, we looked for any peaks in L_50_ around dawn, potentially associated with the dawn chorus. At all sites, we observed relatively high L_50_ values within 1 hr of sunrise (Fig. 2); thus, we limited further acoustic index analysis to 0.5 h before and 2.5 h after local sunrise. Sunrise times were calculated from the US Navy Astronomical Applications Department database (http://aa.usno.navy.mil/data/docs/RS_OneYear.php).

To identify and remove windy and rainy conditions or any recordings with anthropogenic noise, we visually searched through flat‐weighted 1‐sec 1/3 octave band daily spectrograms plotted using the Acoustic Monitoring Toolbox (AMT; developed by the National Park Service 2013). We excluded recordings where >25% were obscured by wind, rain, or anthropogenic noise. To further exclude anthropogenic sounds and isolate avian activity, we limited acoustic analyses to frequencies above 1250 Hz (Brumm and Slabbekoorn 2005).

We calculated ACI, a bioacoustic index known to be correlated with the number of vocalizations produced by a bird community (Farina et al. 2011b; Pieretti et al. 2011; Farina and Pieretti 2014). The ACI was calculated as follows:

(1) $$\text{ACI}{}_{f}=\frac{\sum_{t=1}^{n}\left|\text{SPL}{}_{t}-\text{SPL}{}_{t+1}\right|}{\sum_{t=1}^{n}\text{SPL}{}_{t}},$$

where the absolute differences in SPL between adjacent seconds (*t*) within a 1/3 octave band (f) were summed for the entire 3‐hr period around sunrise (*n*) and divided by the total SPL of the octave band around sunrise. The resulting ACI values for each 1/3 octave band (*N* = 8 octave bands between 1250 and 6300 Hz) were then summed to generate a single ACI value for each day:

(2) $$\text{ACI}{}_{\mathrm{tot}}=\sum_{f=1}^{N}\text{ACI}{}_{f}.$$

One sec 1/3 octave band ACI calculations were performed in MATLAB 2014a (The MathWorks Inc., Natick, MA).

To calculate ACI at a finer temporal and spectral resolution using the original audio files in mp3 format, we used the *seewave* package in program R version 3.2.2 (Sueur et al. 2008a; R Core Team 2015). We found finer temporal and spectral resolution ACI of limited utility for our research objectives (see Appendix S2 for detailed results); thus, hereafter, we focus on the results from the daily 1‐sec 1/3 octave band resolution ACI calculated over the 3 h around sunrise.

To examine the effect of meteorological parameters on ACI values, we obtained wind speed, temperature, and humidity data from the National Oceanic and Atmospheric Administration weather buoy no. BLTA2 in Bartlett cove (58°27′18″N 135°53′20″W).

### Species‐specific acoustic analysis

To ensure that the ACI was capturing actual changes in songbird acoustic activity, we compared values with call rates of three species present in spring recordings at Bartlett Cove. Species were selected based on call abundance:

1. Varied thrush** **(*Ixoreus naevius*) – common breeders in coniferous forests from Alaska to northern California, identified as useful indicators of forest health (Rosenberg et al. 2003). In southeast Alaska, where the density of winter residents is low, the arrival of migrants in early April is easily distinguished (George 2000). Varied thrush song is a simple, distinctive whistled tone on a single pitch, varying from 2500–5850 Hz.
2. Pacific wren (*Troglodytes pacificus*) – recently separated from the winter and Eurasian wren (*Troglodytes hiemalis*) based on its distinct song (Toews and Irwin 2012). Pacific wren arrive at Alaskan breeding grounds between April and May and some shift southwesterly to wintering grounds in British Columbia and Washington in September–December (Toews and Irwin 2012). Birds are associated with old‐growth forests or, on the coast, riparian areas enriched with salmon‐derived nutrients. Pacific wren are known for their long, complex vocalizations; their songs comprise complex note arrangements and high frequency modulations (3–9 kHz; Toews and Irwin 2008).
3. Ruby‐crowned kinglet (*Regulus calendula*) – is a small songbird whose peak spring migration occurs in mid‐to‐late April, with birds returning to wintering grounds in the southern US to Mexico, between late September and early October (Sinclair et al. 2003). Both female and male ruby‐crowned kinglets sing, the latter producing complex and rich song primarily in the breeding season, but also during migration and on wintering grounds (Swanson et al. 2008).

We used sound analysis software Raven Pro 1.5 (Cornell University, Ithaca, NY) to develop automated detectors for each species call (details provided in Methods S1). In order to load sound files into Raven, we first converted mp3 to wav format using the AMT (National Park Service 2013). We converted the first 20 sec of each 2‐min recording from 3 am to 9 am, to subsample around sunrise from March to July. We used band‐limited energy detectors, also known as time–frequency energy detectors, which are detection algorithms used to search for and identify calls of interest within recordings. To define detector specifications, including signal‐to‐noise ratio (SNR) thresholds, frequency bands, and time intervals, we used the properties of a random subset of each species' calls selected within our recordings (Appendix S1, Table S1.1). We identified and removed all false positives by visually checking each detection using Raven's selection review feature (details of time and frequency scales are provided in Table S1.1). We then visually scanned a random subset (15–20%) of each year's recordings to search for false negatives. We summarized the number of true detections by date and corrected the total by applying the annual false‐negative rate (Table S1.2).

### Species diversity acoustic analysis

To evaluate the relationship between ACI values and acoustic avian species diversity, we estimated the number of each species call and song within a subset of recordings. We converted the first 30 sec of each 1‐min recording from 4 am to 7 am of a random subsample of 12 days in 2014 from mp3 to wav. We used Raven to visualize recordings (settings: brightness 49, contrast 58, and spectrogram window size 678). We counted all visible vocalizations of each species, noting different vocalization types of the same species (e.g., Dark‐eyed Junco, *Junco hyemalis*, song vs. chip note). If we were unable to identify a call to species, we labeled it with a unique identifier (i.e., “pseudo‐species”) and saved the spectra in a call library. All vocalizations with similar spectra were labeled with the same unique identifier.

To calculate species vocalization diversity indices, we used the package *vegan* in R (Oksanen et al. 2013). We calculated Shannon diversity, Simpson diversity, and species richness (Hurlbert 1971).

### Statistical analysis

To identify significant changes in ACI values during the spring and fall migration period, we used a Bayesian change point analysis (Barry and Hartigan 1993). Bayesian change point (BCP) analysis calculates the posterior probability that any given point in the time series is an abrupt change. We used the package “bcp” in R (Erdman and Emerson 2007). We used 10,000 iterations of a Markov Chain Monte Carlo sampler after a burn‐in of 5000 iterations. We define a “high” probability of change as >0.5.

To determine the relationship between ACI and corrected detections of each species bird calls, temperature, humidity, and wind speed, we used general linear mixed models, each with a Gaussian error structure and inverse link (Bolker et al. 2009). We fitted four models: three including the number of vocalizations of each species, respectively; and one with temperature, humidity, and wind speed as continuous fixed variables. In all models, we used date as a continuous random variable to remove the effect of daily environmental conditions. Models were fitted using the “lme4” package in R (Bates et al. 2012), and all results are presented as mean ± standard error unless noted otherwise. To determine whether ACI values were related to the diversity of vocalizing avian species, we fit three general linear models: with Simpson diversity, Shannon diversity, and species richness as independent variables.

## Results

**Figure 3:**
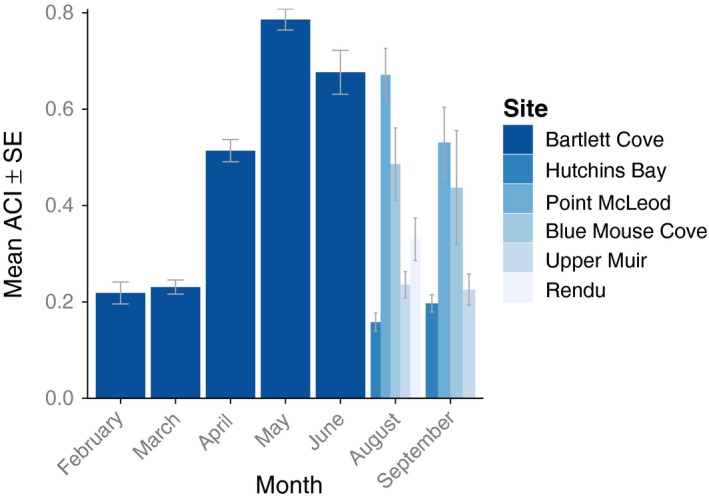
Mean Acoustic Complexity Index values (±standard error) for each month summarized over six sites in Glacier Bay, Alaska.

We found the highest mean ACI at Bartlett Cove (0.56 ± 0.02) and at Point McLeod (0.60 ± 0.05). In Bartlett Cove, we found the highest mean ACI in 2014 (0.71 ± 0.03), and lowest in 2012 (0.59 ± 0.03). Among years and sites, we found that ACI values peaked from late spring (April) to summer (June; Fig. 3).

### Soundscape phenology

**Figure 4:**
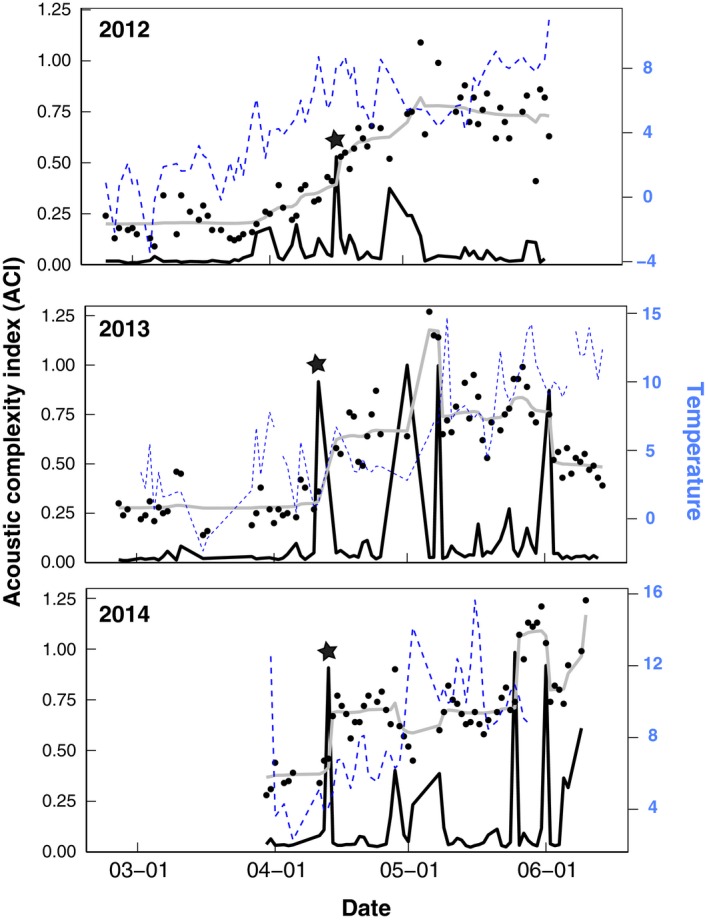
The *y*‐axis represents the probability of a change (*black line*) in the estimated mean ACI (*gray line*) at Bartlett Cove, Glacier Bay, Alaska, in 2012, 2013, and 2014. Black dots represent the daily ACI values calculated around sunrise. Black stars represent the first spring peak in ACI > 0.5, likely representing spring transition. Dashed blue lines represent mean daily temperature.

In spring recordings at Bartlett Cove, we found the first peak in probability of change in ACI values occurred on April 16, April 11, and April 13 in 2012, 2013, and 2014, respectively (Fig. 4; probability of change 0.53, 0.92, 0.91). On these days with high probability of change, ACI was 2.2, 2.1, and 1.8 times higher than the mean ACI of previous days. This change in ACI corresponds within 10 days of the first peak in songbird abundance reported on all eBird checklists within the Skagway–Hoonah–Angoon region (encompassing Glacier Bay National Park; eBird 2012). Changes in ACI were also related to temperature (*Z*_1_ = 0.03, 95% confidence intervals: 0.03–0.04, Figure 4) and weakly to pressure (*Z*_1_ = 0.004, 95% confidence intervals: <0.001–0.008).

**Figure 5:**
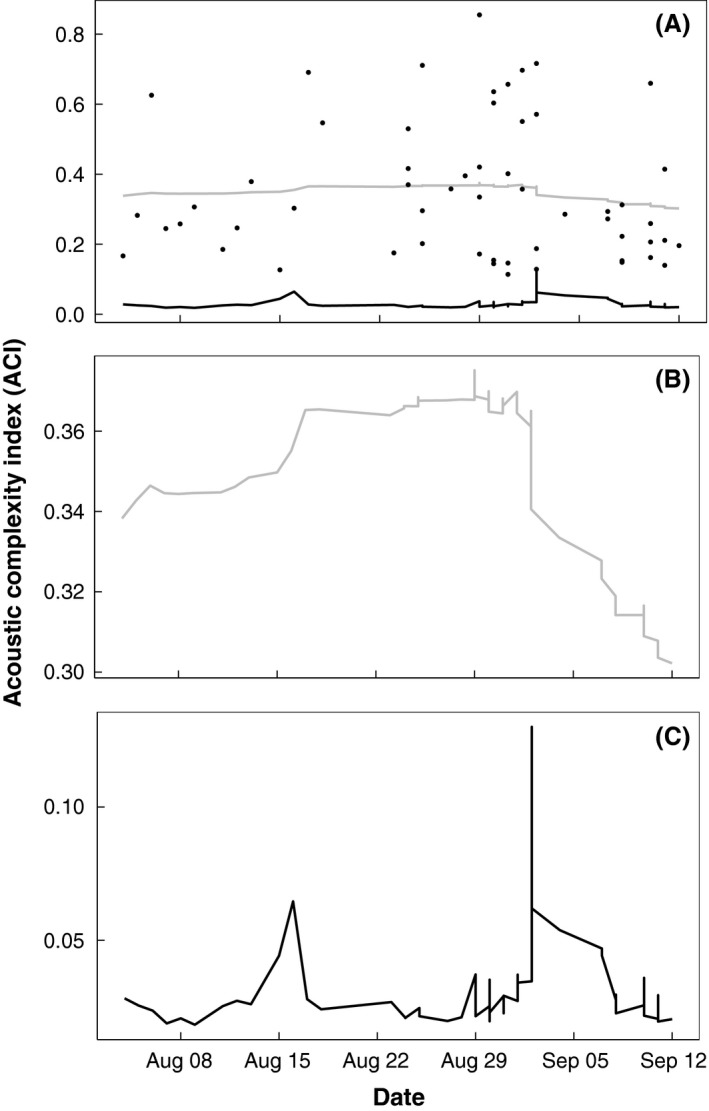
The *y*‐axis represents the probability of a change (*black line,* (A) *and* (C)) in the estimated mean ACI (*gray line,* (A) *and* (B)) among five sites (Hutchins Bay, Point McLeod, Blue Mouse Cove, Upper Muir, and Rendu) in Glacier Bay, Alaska, during 2011. Black dots in (A) represent the daily AIC values calculated around sunrise.

We found a higher percentage of days obscured by wind and rain in fall recordings (64.51 ± 0.08%) versus spring (29.62 ± 0.03). Wind and rain resulted in 0–20 usable days at each site in the fall. Therefore, we combined all sites to examine changes in fall ACI. We found a low probability of change in ACI between August and September (Fig. 5A). However, there was a small, but marked decline in ACI beginning September 2 (Fig. 5B,C).

### Soundscape indices and species abundance

**Figure 6:**
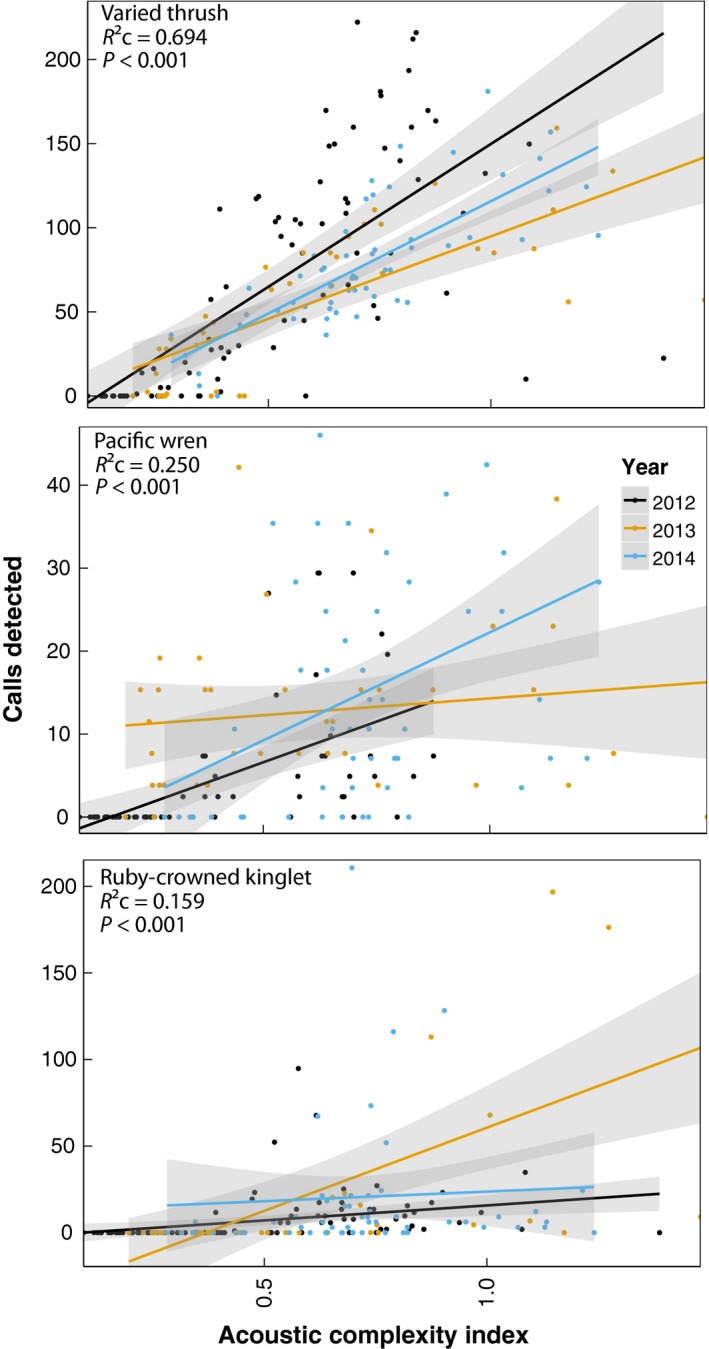
The relationship between the number of songbird calls detected (corrected for false‐negative rates) and ACI values. $R_{c}^{2}$ values indicate *R* ^2^ values conditional on year, included as a random factor.

For all three species, ACI was positively related to the total number of call detections (all *P *<* *0.001; varied thrush *Z*_1_ = 58.16, $R_{c}^{2}$ = 0.69; Pacific wren *Z*_1_ = 9.76, $R_{c}^{2}$ = 0.25; ruby‐crowned kinglet *Z*_1_ = 28.50, $R_{c}^{2}$ = 0.16; Fig. 6). The relationship between ACI and calls detected was strongest for varied thrush (parameter estimate ± standard error: 2.43 ± 0.04). ACI was also positively related to richness (*P *=* *0.01, Parameter estimate ± SE = 0.022 ± 0.007, *R* ^2^ = 0.50) and weakly with diversity (Shannon: *P *=* *0.06, Parameter estimate ± SE = 0.25 ± 0.12, *R* ^2^ = 0.32; Simpson: *P *=* *0.10, Parameter estimate ± SE = 0.72 ± 0.40, *R* ^2^ = 0.24) of species vocalizations in recordings.

## Discussion

Acoustic recordings are an increasingly effective technique for monitoring terrestrial and aquatic environments, where soundscape ecology is a rapidly growing field of research (Sueur et al. 2008b; Pijanowski et al. 2011a; Gasc et al. 2013; Towsey et al. 2014a). We examined the application of acoustic recording and bioacoustic analysis to examine patterns in songbird phenology. We found that the ACI, an acoustic index, calculated on 1‐sec 1/3 octave band spectra successfully identified changes in bioacoustic activity at spring transition when migratory songbirds arrive in GBNP, Alaska. ACI was positively related to three potential ecosystem health indicator species and richness of species vocalizations. We conclude that acoustic recording paired with rapid acoustic indices could be a cost effective way of monitoring changes in bird song phenology as a result of global climate and land‐use change.

### ACI as an indicator of spring and fall phenology

We show that abrupt changes in ACI calculated using 1‐sec 1/3 octave band spectra (hereafter, ACI) correspond with estimates of spring transition and the abundance of migrant songbird species. Monitoring shifts in songbird migration has been suggested as a useful indicator of climate‐driven changes in spring phenology, where a growing number of studies indicate that migratory birds are arriving successively earlier at breeding grounds (Root et al. 2003; Crick 2004; Lehikoinen et al. 2004). However, the scale and complexity of spring migration and the infeasible logistics of current monitoring techniques have precluded measurements of songbird phenology among species at large scales. Acoustic recording can be implemented at massive scales, and indices are an effective method of extracting meaningful information from resulting datasets. Using 1‐sec 1/3 octave band, acoustic data requires less‐intensive storage and computation capacity than wav or mp3 format. Moreover, the calibration method used provides absolute measures of sound levels necessary to draw meaningful comparisons of habitats through time and at different locations (Mennitt and Fristrup 2012; Merchant et al. 2015). Thus, ACI calculated at this resolution is a fast and relatively precise method of evaluating the arrival of songbirds at multiple spatiotemporal scales, independent of the type of acoustic equipment.

ACI values were less sensitive to changes in fall soundscapes. The relationship between climactic variables and phenology of fall migration is highly variable and inconclusive between and among species (Sparks and Mason 2004; Sparks et al. 2007; Van Buskirk et al. 2009). Also, the detectability and emission of vocalizations declines throughout the breeding season, as birds secure territories and raise offspring, making any shift in ACI difficult to interpret (Best 1981; Selmi and Boulinier 2003). Furthermore, the large number of recording days obscured by weather events resulted in little usable data at any individual site; thus, to increase sample size, we combined recordings from all sites. Natural habitat‐driven spatiotemporal differences among sites in Glacier Bay may have resulted in high variability, obscuring significant patterns in songbird communities within combined data. Whether the lack of robust change in ACI in the fall was due to more subtle declines in complexity of late summer soundscapes or more frequent weather events, ACI is likely a less‐effective method of indicating fall songbird migration phenology.

### Relationship between ACI and vocalizations

ACI values were positively related to song detection of three songbird species present in GBNP (Fig. 6): varied thrush, which have a simple whistled song on a single pitch, but are the most common bird we recorded in the park; ruby‐crowned kinglet, which were also common, but have a more complex call; and Pacific wren, which were less common in our recordings, but have a remarkably complex call (Toews and Irwin 2012). ACI was most strongly related to varied thrush song detections and showed the weakest relationship with pacific wren song detections. This pattern is likely due to the 1‐sec resolution at which we calculated ACI; at this scale, frequent and irregular pulses of a whistle at slightly different pitches are driving the complexity of the soundscape. Finer resolution ACI values were weakly related to all three species vocalizations; however, the strongest relationship was with pacific wren song detections (*R* ^2^ = 0.13, Table S2.1). Pacific wren have the most complex call of the three species; thus, the finer resolution temporal steps were likely able to capture intrasong frequency modulation.

ACI values were also positively related to the richness of species vocalizations in Glacier Bay recordings. Similarly, previous studies have linked acoustic indices to avian species richness (Towsey et al. 2014b) and the number of bird vocalizations (Pieretti et al. 2011). Because ACI is related to both species richness and varied thrush song abundance, we postulate that varied thrush may act as an indicator of the complexity of the Glacier Bay soundscape. Because varied thrush are sensitive to the structure, productivity, and composition of temperate forests, they are considered good indicators of forest ecosystem health (Hilty and Merenlender 2000). Consequently, recordings with higher ACI values may also indicate the presence of healthy stands of forest.

### ACI as an effective large‐scale ecological monitoring technique

One of the greatest advantages of combining acoustic recording and soundscape assessment is the speed and efficiency of extracting meaningful information from long recordings over a large spatial scale. Although recordings of songbird calls have been used previously to estimate abundance and species richness, acoustic analysis typically involved training observers to visually scan spectrograms or the construction of large numbers of automated call detectors (e.g., band‐limited energy detectors; Swiston and Mennill 2009). Manually scanning spectrograms is an arduous process, requiring teams of trained sound analysts to sift through massive datasets, while the construction of good‐quality detectors requires significant amounts of training data (Towsey et al. 2012; Sanders and Mennill 2014).

Calculation of ACI was relatively straightforward, and with newly available open source software capable of calculating numerous acoustic index types, analysis will only become faster and more user‐friendly (Sueur et al. 2008a; Villanueva‐Rivera and Pijanowski 2015). Although calculations are straightforward, we caution that the duration the of time and frequency range an index is calculated over must be carefully considered to ensure the indices are capturing meaningful or intended characteristics of a soundscape. To capture songbird activity, we recommend limiting acoustic index analysis to sunrise, capturing the dawn chorus, and to frequencies that capture biophony, or natural biological sounds produced by vocalizing animals (i.e., approximately 1500–8000 Hz; Krause 1998; Kasten et al. 2012).

### Further potential applications of ACI

ACI may also correspond with other relevant biological patterns. For example, ACI was highest in 2014, which was an irruption year for varied thrush (eBird 2012). Many boreal bird species show marked variation in abundance (i.e., irruptions), which is thought to be related to cyclical food availability (Lack 1954). During irruption years, larger numbers of varied thrush are observed at wider ranging distributions, and individuals spend longer periods at breeding grounds (Wells et al. 1996). However, little is known about why these events occur and whether their frequency will be affected by climate change. Another potential application of acoustic recording and analysis of biophony is to examine range shifts or extinctions of songbird species (Greenville et al. 2012). Climate change is expected to shift the range and threaten the status of over half of North American bird species (National Audubon Society 2014). Mapping predicted shifts in bioacoustic activity at a landscape level could provide valuable information for conservation management. For example, mapping of anthrophony (any acoustic signal created by human activities; Gage et al. 2001) at a continental scale in the United States has provided more extensive information for managing noise within national parks (Mennitt et al. 2014).

Finally, acoustic indices have been linked to broad ecosystem dynamics, habitat types (Pekin et al. 2012), and biodiversity (Sueur et al. 2008b). Thus, we argue that modern acoustic technology paired with rapid assessment of recordings could not only determine shifts in songbird phenology due to climate change, but could subsequently elucidate large‐scale ecosystem‐level repercussions (Sueur et al. 2014; Towsey et al. 2014a; Merchant et al. 2015). As the phenology, range, and composition of animal communities continues to change, and as research develops, soundscape analysis could be a valuable coarse, large‐scale monitoring tool of an entire ecosystem or community.

### ACI resolution

ACI is the average difference in SPL values between frequency bins and temporal steps, generally calculated using a msec temporal resolution (Farina et al. 2011b; Pieretti et al. 2011; Pieretti and Farina 2013). In our analyses, we used ACI from calibrated acoustic data (1‐sec temporal steps and 1/3 octave band frequency bins), resulting in a coarser time and frequency resolution than relative levels of signal amplitude (Mennitt and Fristrup 2012).

We found fine‐resolution ACI (0.01‐sec temporal resolution and 86.1 frequency resolution) to be less useful for indicating the richness of species vocalizations within recordings and the abundance of common songbirds. Conversely, coarse‐resolution ACI was strongly related to the most common songbird call abundance and the diversity of species vocalizations. Most vocalizations in our recordings were 0.2–1 sec in duration. Thus, high ACI values calculated using 1‐sec 1/3 octave band spectra are likely driven by the comparison between a species call in a one‐second temporal step and silence, another syllable, or another species call in an adjacent temporal step (Fig. S3.1). With an increase in call abundance, especially of many different species, coarse‐resolution ACI will be higher, due to an increased probability that one‐second temporal steps differ. With a decrease in call abundance, coarse‐resolution ACI will be lower, due to an increased probability that temporal steps are both silent, and thus, the same.

We caution that ACI calculated at a 1‐sec temporal resolution may be less useful for recordings with single species vocalizing continuously or with high levels of anthropogenic background noise. Vocalizations in dawn chorus recordings in GBNP were relatively sparse, with few of the same species calling for multiple seconds repeatedly (see Fig. S3.1 for typical dawn spectrograms). Moreover, there was little anthropogenic noise at these remote sites, and days with noise were removed from ACI analyses. Our results highlight the importance of considering the temporal and spectral resolution of recordings for acoustic index calculation. Varying these parameters will result in measurements of different components of the acoustic environment.

## Conclusions

The urgent need to mitigate the loss of biodiversity highlights the need to quantify ecological changes at large spatiotemporal scales (Dornelas et al. 2013). With rapid rates of projected climate change, the need to monitor shifts in phenology of key species has become particularly important (Heller and Zavaleta 2009). Acoustic ecology research and the associated analytical tools are emerging as an invaluable way to monitor shifts in phenology and community assembly due to climate change. As links are made between soundscape complexity and ecosystem functioning, even more comprehensive information will be extractable from acoustic recordings. Acoustic ecology has earned a place in the important endeavor of identifying and predicting changes in biological communities which are vital for adaptive and effective global conservation management.

## Conflict of Interest

None declared.

## Supporting information


**Appendix S1.** Band‐limited energy detector specifications.
**Appendix S2.** Finer resolution ACI calculations.
**Appendix S3.** Dawn chorus spectrogram example.
