# Supplementary material for: Using bioacoustics to examine shifts in songbird phenology
Source: Ecol Evol. 2016 Jun 12;6(14):4697–710. doi: 10.1002/ece3.2242 (PMC4979700; doi:10.1002/ece3.2242)
Supplement: Supplementary file 1 — Appendix S1. Band‐limited energy detector specifications. Appendix S2. Finer resolution ACI calculations. Appendix S3. Dawn chorus spectrogram example. [file ECE3-6-4697-s001.docx]

**Using bioacoustics to examine shifts in songbird phenology**

**Ecology and Evolution**

**Supplementary Materials**

# Appendix 1: Band-limited energy detector specifications

To specify the acoustic and temporal patterns of band-limited energy detectors we listened to recordings and searched visually in spectrograms for each species song. Once potential songs were selected and saved, we verified their identity using Cornell University’s Macauley Library (Cornell University 2015) and expert advice. Once we confirmed song structure we took the following measurements on a random subset of 10-15 songs by selecting songs in Raven Pro 1.5 (Cornell University, Ithaca, New York): minimum frequency, maximum frequency, song duration, and signal to noise ratio. We then constructed band-limited energy detectors for each species using the average of each measurement. We ran the detectors with diagnostics enabled to calculate occupancy and fine tune each parameter to maximize the number of songs detected. The final parameters for each species detector are presented in Table S1.1. We manually reviewed and removed all false positives (e.g. other sounds mistakenly identified as the song of interest) using Raven’s selection review feature. To calculate false negative rates we counted the actual number of each species song in 12-20 recordings each year (Table S.1.2). We corrected each species song detections for annual false negative rate.

To ensure false-negative corrected detections had a similar relationship with ACI (Fig. 6, main text) as actual song counts, we used general linear mixed models comparing ACI with actual song counts (above) included as a fixed variable. Models were fitted in program R version 3.2.2 using the *lme4* package and in each case date was added as a random variable (Bates et al. 2012; R Core Team 2015). We found that corrected R^2^ values are slightly lower for varied thrush and pacific wren, and slightly higher for ruby-crowned kinglet when analyzing actual calls (Table S1.3). However, the trends are similar, where ACI still has the strongest positive relationship with varied thrush (R_c_^2^ = 0.5).

LITERATURE CITED

Bates, D., M. Maechler, and B. Bolker (2012). lme4: linear mixed-effects models using S4 classes. R package version 1.1-6. <http://CRAN.R-project.org/package=lme4>. CRAN.

Cornell University (2015). Macauley Library. Cornell lab of Ornithology, Ithaca, USA. [www.macaulaylibrary.org](http://www.macaulaylibrary.org), Access date: June 2015

R Core Team (2015). R: a language and environment for statistical computing. R Foundation for Statistical Computing, Vienna, Austria. URL: <http://www.R-project.org/>.

**Table S1.1:** Band-limited energy detector parameters for each species song, created in Raven Pro 1.5 (Cornell University, Ithaca, New York).

|  | **Min freq** | **Max freq** | **Min duration (sec)** | **Max duration (sec)** | **Min occupancy** | **SNR threshold (dB)** | **Exclusion band (Hz)** | **Exclusion band SNR (dB)** |
| --- | --- | --- | --- | --- | --- | --- | --- | --- |
| Varied Thrush | 2540 | 3700 | 0.699 | 1.997 | 60 | 3 | n/a | n/a |
| Winter Wren | 3800 | 6300 | 2.014 | 10.003 | 20 | 5.5 | 1500-3800 | 0 |
| Ruby-crowned Kinglet | 2650 | 6300 | 1.298 | 2.896 | 55 | 2 | 2930-3200 | 10 |

**Table S1.2:** False negative rates of band-limited energy detectors for each species song in each year. False negatives occur when detectors fail to identify the call of interest. Note: all false positives were removed and subsequent song detections were corrected for annual false negative rates before further analyses.

| **Species** | **Year** | **False Negative rate** |
| --- | --- | --- |
| Varied thrush | 2012 | 0.16 ± 0.03 |
|  | 2013 | 0.12 ± 0.04 |
|  | 2014 | 0.17 ± 0.05 |
| Winter wren | 2012 | 0.52 ± 0.07 |
|  | 2013 | 0.65 ± 0.05 |
|  | 2014 | 0.62 ± 0.07 |
| Ruby-crowned kinglet | 2012 | 0.44 ± 0.10 |
|  | 2013 | 0.47 ± 0.16 |
|  | 2014 | 0.26 ± 0.10 |

**Table S1.3:** General linear mixed model results examining the relationship between 1 sec 1/3 octave band ACI and actual counts of each species song. We counted calls of each species on a subset of days (12-20 days per year) to calculate false negative rates of band-limited energy detectors. We show model parameter estimates and their 95% confidence intervals (CI) and conditional R squared values (R_c_^2^ adjusted for the random variable, date).

| **Species** | **N** | **Parameter Estimate** | **2.5% CI** | **97.5% CI** | **R_c_^2^** |
| --- | --- | --- | --- | --- | --- |
| Varied thrush | 47 | 2.07 | 1.91 | 2.23 | 0.50 |
| Pacific wren | 52 | 0.52 | 0.11 | 0.93 | 0.18 |
| Ruby-crowned kinglet | 48 | 4.41 | 4 | 4.82 | 0.39 |

# Appendix 2: Finer resolution ACI calculations

To achieve a finer temporal and spectral resolution for ACI calculation an FFT was performed using a non-overlapping window length of 512 samples and a Hamming window. The resulting FFT spectra had a time resolution of 0.01 sec (*t* in Eq. 1, main text) and frequency bin resolution of 86.1 Hz. Unlike the coarser resolution, it was not possible to produce calibrated SPLs. Due to computational constraints we were unable to calculate fine-scale ACI on the entire 3 hour period around sunrise, thus we split recordings into 30 minute clips and took a daily average of ACI. To ensure ACI values calculated at this resolution were comparable with our coarser 1/3 octave band resolution we limited fine-scale ACI analysis to frequency bins from 1,250-6,300 Hz.

To determine if ACI calculated at a finer resolution was better suited to quantify avian species in the acoustic environment, we assessed the relationship of fine-scale ACI with the corrected detections of bird calls and coarser resolution ACI values. We used four separate general linear mixed models, each with a Gaussian error structure and inverse link: three including the number of vocalizations of each species respectively; and one with 1 sec 1/3 octave band resolution ACI as continuous fixed variables. In all models we used date as a continuous random variable to remove the effect of daily environmental conditions. Models were fitted using the ‘lme4’ package in R (Bates et al. 2012). To determine if finer resolution ACI values were better suited to examine the diversity of vocalizing avian species, we fit three general linear models: with Simpson diversity, Shannon diversity, and species richness as independent variables.

We found weak evidence of a relationship between fine-scale ACI and species call abundance (R^2^ = 0.03-0.13, Table S2.1, Fig. S2.1); coarser resolution ACI (R^2^ = 0.11-0.32, Table S2.2); and species richness or diversity (R^2^ = 0-0.02, Table S2.3).

**Table S2.1:** Results of general linear mixed models comparing the abundance of each species call detections (with false positives removed, corrected for annual false negative rate) with fine-resolution-ACI values. We show model parameter estimates and their 95% confidence intervals (CI) and conditional R squared values (R_c_^2^ adjusted for the random factor, date).

| **Species** | **Parameter estimate** | **2.5% CI** | **97.5% CI** | **R_c_^2^** |
| --- | --- | --- | --- | --- |
| Varied thrush | -0.3 | -0.83 | 0.24 | 0.04 |
| Pacific wren | -0.03 | -0.15 | 0.09 | 0.13 |
| Ruby-crowned kinglet | 0.07 | -0.34 | 0.48 | 0.03 |

Table S2.2: Results of general linear mixed models comparing ACI calculated in 1 sec time steps with fine-scale-ACI values. Date was included as a random factor. We show model parameter estimates and their 95% confidence intervals (CI) and conditional R squared values (R_c_^2^ adjusted for the random factor, date).

| **Parameter Estimate** | **2.5% CI** | **97.5% CI** | **R_c_^2^** |
| --- | --- | --- | --- |
| 0.001 | -0.0002 | 0.002 | 0.11 |

Table S2.3: Results of linear models comparing three diversity indices (species richness, and Simpson and Shannon diversity) with finer-resolution-ACI values in a subset of recordings in 2014. We show model parameter estimates and their 95% confidence intervals (CI) and R squared values.

| **Index** | **Parameter estimate** | **2.5% CI** | **97.5% CI** | **R^2^** |
| --- | --- | --- | --- | --- |
| Species richness | -0.21 | -1.36 | 0.93 | 0.02 |
| Simpson diversity | -0.21 | -1.36 | 0.93 | 0.02 |
| Shannon diversity | -1.62 | -17.71 | 14.47 | 0.01 |

**Figure S2.1:** The relationship between the number of songbird songs detected (with false positives removed, corrected for false negative rates) and finer-scale ACI values.
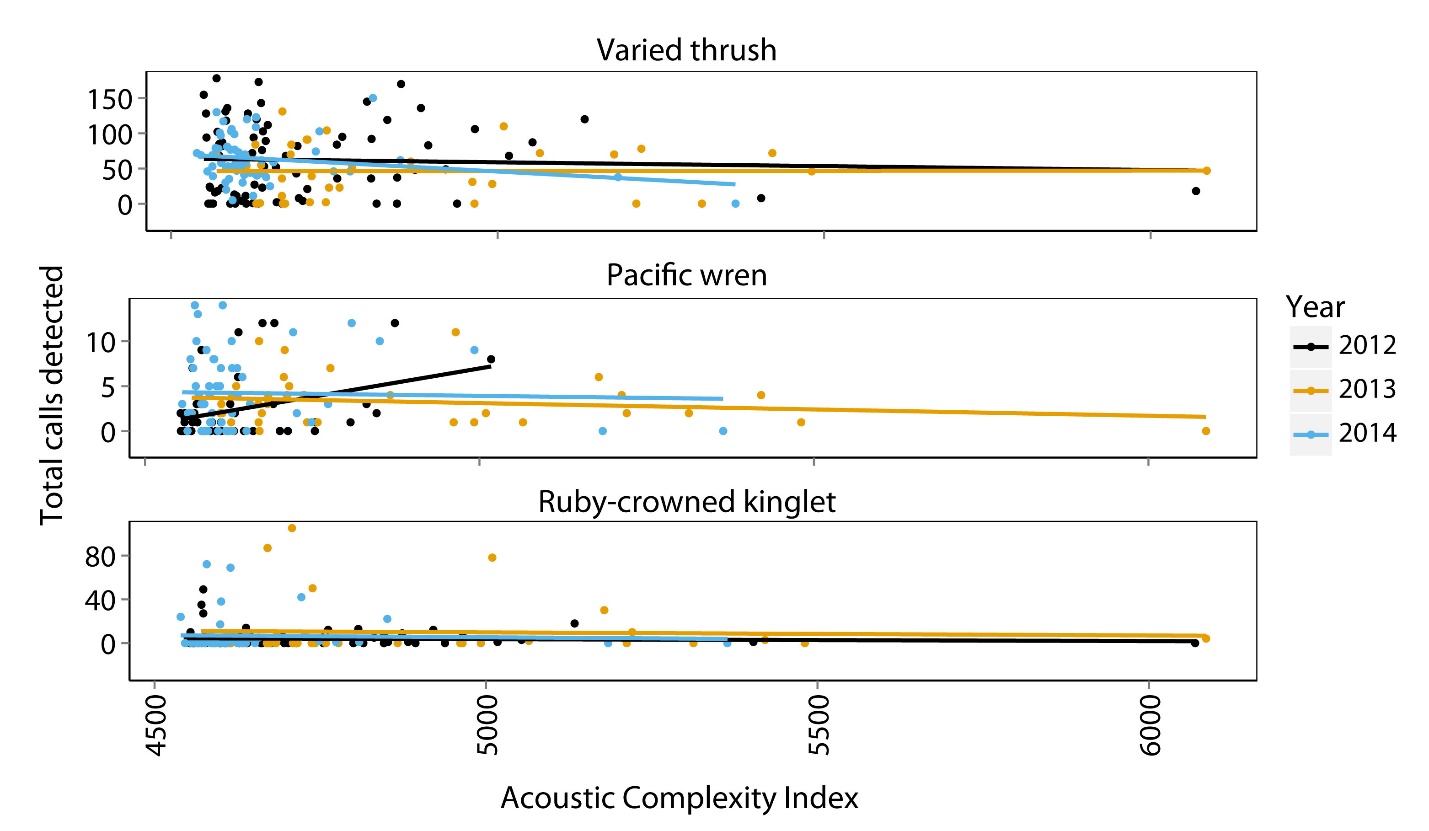


# Appendix 3: Dawn chorus spectrogram example

**Figure S3.1**: Spectrogram produced in Raven Pro 1.5 (brightness 49, contrast 58, and spectrogram window size 678) on April 17, 2014 at 0500 am (A) and May 25, 2014 at 0400 am (B). Red vertical lines delineate the first 6 second increments (temporal step size of ACI calculated using 1 sec 1/3 octave band spectra; *t* in Eq. 1 of main text) and green horizonal lines represent the lower and upper frequency limits of ACI calculations.


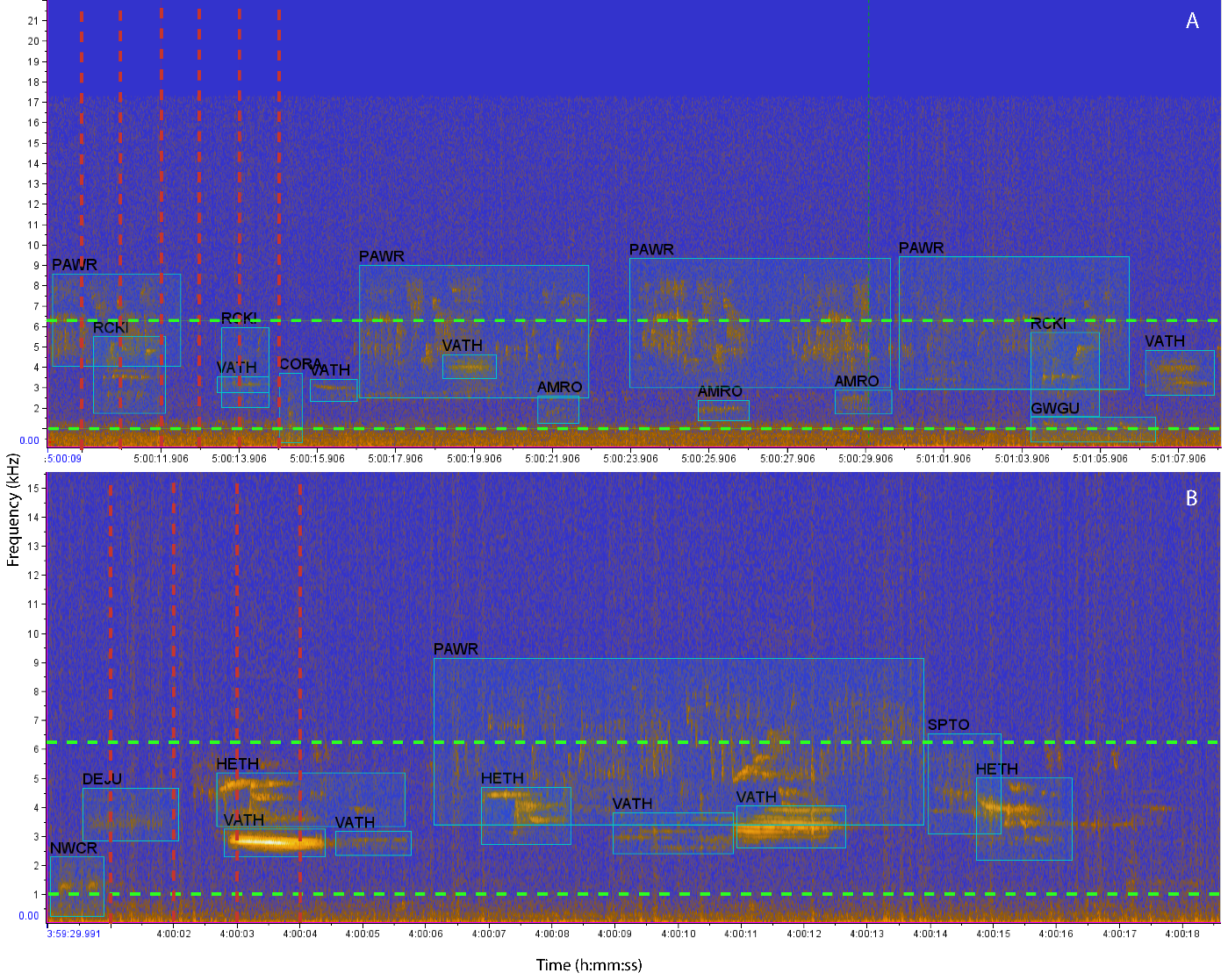


Common species observed in Bartlett Cove, Glacier Bay spring recordings –

PAWR: Pacific wren (*Troglodytes pacificus*)

RCKI: Ruby-crowned kinglet (*Regulus calendula*)

VATH: Varied thrush (*Ixoreus naevius*)

CORA: Common raven (*Corvus corax*)

AMRO: American robin (*Turdus migratorius*)

GWGU: Glaucous-winged gull (*Larus glaucescens*)

NWCR: Northwestern crow (*Corvus caurinus*)

DEJU: Dark-eyed junco (*Junco hyemalis*)

HETH: Hermit thrush (*Catharus guttatus*)

SPTO: Spotted towhee (*Pipilo maculatus*)
